# Assessment of left ventricular volumes and primary mitral regurgitation severity by 2D echocardiography and cardiovascular magnetic resonance

**DOI:** 10.1186/1476-7120-11-46

**Published:** 2013-12-27

**Authors:** Caroline M Van De Heyning, Julien Magne, Luc A Piérard, Pierre-Julien Bruyère, Laurent Davin, Catherine De Maeyer, Bernard P Paelinck, Christiaan J Vrints, Patrizio Lancellotti

**Affiliations:** 1 Department of Cardiology, University of Liège Hospital, GIGA Cardiovascular Sciences, Heart Valve Clinic, CHU Sart Tilman, Liège, Belgium; 2 Department of Cardiology, University of Antwerp Hospital, Edegem, Belgium; 3 Department of Radiology, University of Liège Hospital, Thoracic and Cardiovascular Imaging, CHU Sart Tilman, Liège, Belgium; 4 Department of Cardiology, University Hospital, Sart-Tilman, B-4000 Liège, Belgium

**Keywords:** Valve, Mitral regurgitation, Left ventricular function, Echocardiography, Cardiac magnetic resonance

## Abstract

**Background:**

Two-dimensional transthoracic echocardiography (2DTTE) remains the first-line diagnostic imaging tool to assess primary mitral regurgitation although cardiovascular magnetic resonance (CMR) has proven to establish left ventricular function more accurately and might evaluate mitral regurgitation severity more reliably. We sought to compare routine evaluation of left ventricular function and mitral regurgitation severity by 2DTTE with assessment by CMR in moderate to severe primary mitral regurgitation without overt left ventricular dysfunction.

**Methods:**

We prospectively included 38 patients (79% of male, age 57 ± 14 years) with at least moderate primary mitral regurgitation, a left ventricular ejection fraction ≥60% and a left ventricular end-systolic diameter ≤45 mm. Patients with evidence of coronary artery disease, arrhythmias or significant concomitant valvular disease were excluded. All patients were scheduled for 2DTTE and CMR.

**Results:**

Left ventricular end-diastolic and end-systolic volumes were significantly underestimated by 2DTTE in comparison with CMR, although there was a strong correlation (Pearson r = 0.81, p < 0.00001 and r = 0.7, p < 0.00001, respectively). Measurement of the regurgitant orifice was similar between 2DTTE PISA method and planimetry by CMR (47 ± 24 vs. 42 ± 16 mm^2^, p = 0.12) with a strong correlation between both imaging techniques (Pearson r = 0.76, p < 0.0001). By contrast, assessment of the regurgitant volume by 2DTTE and by phase contrast velocity mapping by CMR showed poor agreement.

**Conclusions:**

In moderate to severe primary mitral regurgitation without overt left ventricular dysfunction, 2DTTE significantly underestimates left ventricular remodelling in comparison to CMR. Measurement of the regurgitant orifice with planimetry by CMR shows good agreement with the PISA method by 2DTTE and thus may be a valuable alternative to assess mitral regurgitation severity.

## Background

Careful assessment and follow-up of asymptomatic patients with moderate to severe primary mitral regurgitation (MR) is mandatory to define the optimal timing for surgery [1,2]. Echocardiography is recommended as the first-line imaging modality for diagnosing MR and more specifically for establishing its aetiology and mechanism, for quantifying its severity and for evaluating its repercussion on left ventricular (LV) function [3]. Cardiovascular magnetic resonance (CMR) imaging is the method of choice in patients with inadequate echogenicity [4]. Although there are limited data in primary MR, CMR has proved to be a more accurate non-invasive tool than echocardiography to measure LV dimensions [5]. Furthermore, the regurgitant volume (RVol) obtained by phase contrast velocity mapping by CMR has been recently accepted as an accurate non-invasive parameter to evaluate MR severity [6,7]. Planimetry of the anatomic regurgitant orifice has also been validated in a small series of patients and is an attractive new method to assess MR severity [8].

We sought to compare routine evaluation of LV function and MR severity by two-dimensional transthoracic echocardiography (2D TTE) and CMR in patients with asymptomatic moderate to severe primary MR and without overt LV dysfunction.

## Methods

### Study population

The present study concerned 41 prospectively included patients in 2 Belgian centres (CHU Sart Tilman Liège and University of Antwerp Hospital) with at least moderate primary MR (effective regurgitant orifice [ERO] ≥ 20 mm^2^ and/or a RVol ≥ 30 ml) and without overt signs of LV dysfunction or dilatation (LV ejection fraction (EF) ≥60% and LV end-systolic diameter ≤45 mm) after informed consent. Patients with poor echogenicity, history of coronary artery disease, persistent arrhythmias or other significant concomitant valvular heart disease (> mild mitral/aortic stenosis or regurgitation) were excluded. Moreover, we did not enrol patients with CMR-incompatible devices or claustrophobia. The ethical committee of the 2 centres approved the study protocol (Ethisch comité Universitair Ziekenhuis Antwerpen and Comité d'éthique Hospitalo-Facultaire Universitaire de Liège).

### Transthoracic echocardiography

**Figure 1:**
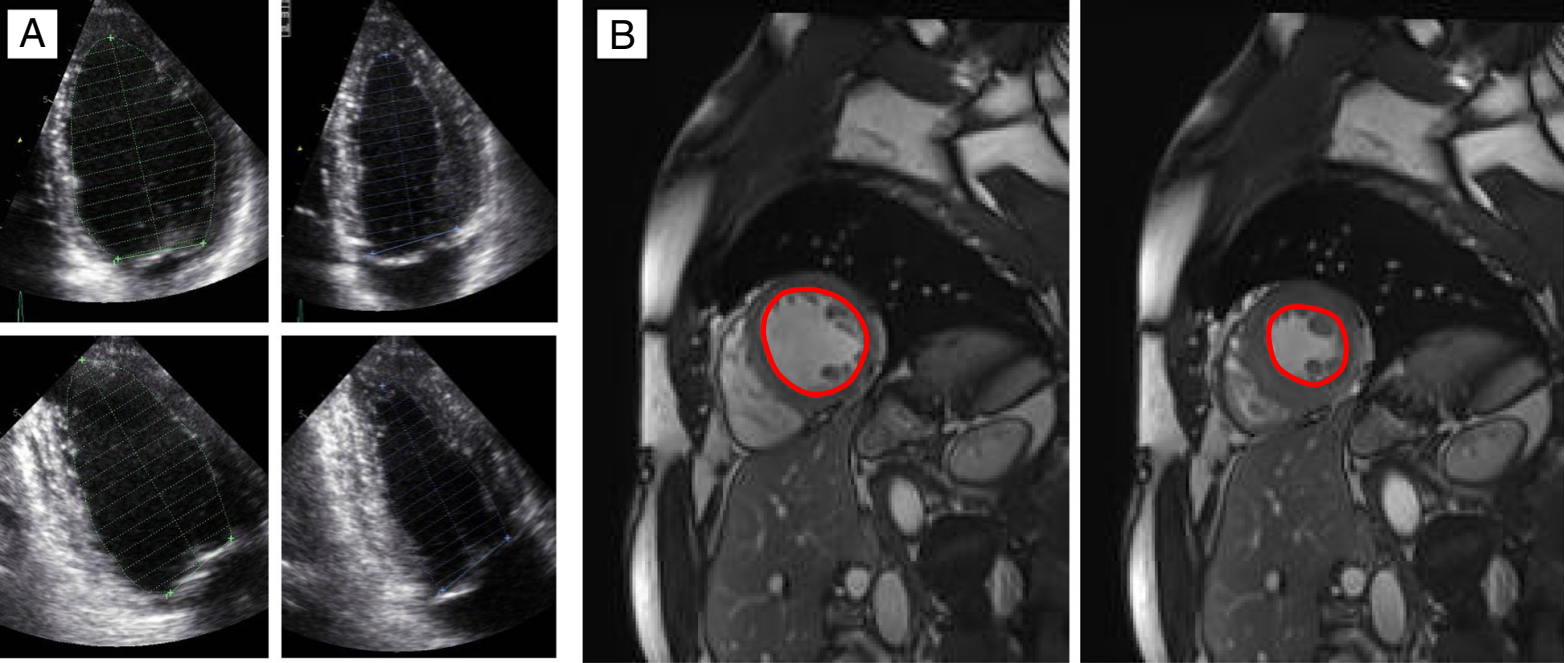
**Measurement of the ejection fraction by 2D TTE and CMR. A**. The LVEF by 2D TTE was obtained by the modified Simpson’s method in the apical four- and two-chamber view. **B**. The LVEF by CMR was calculated by assessment of end-diastolic and end-systolic LV volumes in multiple parallel short-axis slices.

**Figure 2:**
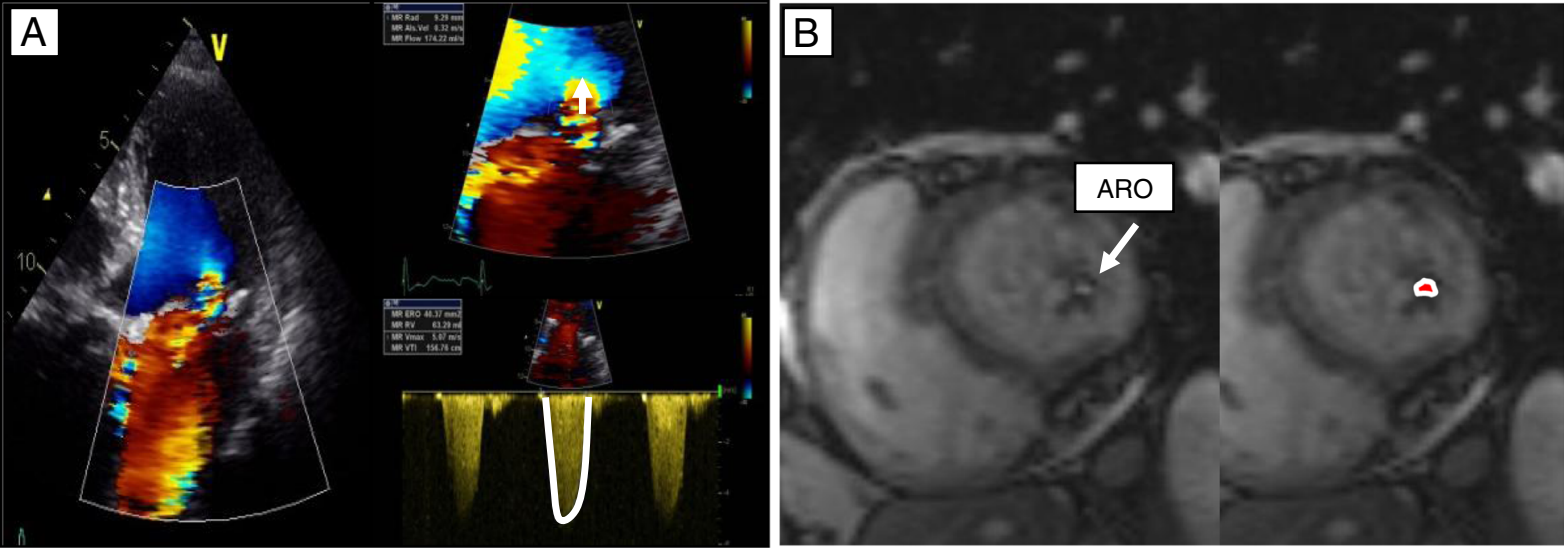
**Measurement of the regurgitant orifice by 2D TTE and CMR. A**. Acquisition of PISA radius and continuous wave Doppler of the MR jet allows calculation of the effective regurgitant orifice. The PISA radius is measured at mid-systole using the first aliasing with a reduced Nyquist limit (15–40 m/s). **B**. The anatomic regurgitant orifice can be measured by planimetry on a slice parallel to the valvular plane obtained by cardiovascular magnetic resonance.

We performed a comprehensive 2D TTE in all patients using Vivid 7 or Vivid 9 cardiovascular ultrasound system (GE Healthcare, Little Chalfont, UK). All data obtained by echocardiography were analyzed off-line with an EchoPAC workstation (GE Vingmed Ultrasound AS, Horten, Norway). End-systolic and end-diastolic LV diameters were measured with M-mode in the parasternal long-axis view according to current recommendations [9] with subsequent calculation of the LVEF by the Teichholz formula. LV volumes and LVEF were quantified by modified Simpson’s method in the apical four- and two-chamber view (Figure 1A). The severity of MR was assessed as recommended [3]. The ERO was quantified using the proximal isovelocity surface area (PISA) method (Figure 2A). RVol was assessed by the PISA method, as well as by the Doppler volumetric method. Moderate primary MR was defined as an ERO between 20 mm^2^ and 40 mm^3^ and/or a RVol between 30 ml and 60 ml while an ERO ≥ 40 mm^2^ and/or a RVol ≥ 60 ml defined severe MR.

### Cardiovascular magnetic resonance

CMR imaging was performed using a 1.5-T scanner (Symphony TIM, Siemens, Erlangen, Germany). Breath-hold ECG-gated steady-state free precession sequences in standard long-axis and multiple parallel short-axis slices were used for the assessment of end-systolic and end-diastolic LV dimensions and volumes (Figure 1B). LV stroke volume (end-diastolic LV volume – end-systolic LV volume) and the LVEF ([end-systolic volume/end-diastolic volume] × 100) were calculated. In 22 patients of the study group, we measured the anatomic regurgitant orifice (ARO) by planimetry of the regurgitant orifice in a slice parallel to the valvular plane and perpendicular to the regurgitant jet at mid-systole as previously described by Buchner *et al.*[8] (Figure 2B). The antegrade LV stroke volume was obtained by phase contrast velocity mapping at the ascending aorta [6]. CMR RVol was calculated as the difference between the LV stroke volume and the antegrade LV stroke volume. All CMR data were assessed by agreement of 2 readers (PJB and LD) who were blinded to echocardiographic data.

### Statistic analysis

All data were analysed with SPSS version 20 (IBM Statistics). Results are expressed as mean ± SD or percentage unless otherwise specified. Comparisons of LV dimensions, ERO/ARO and RVol obtained by 2D TTE and CMR were made using the paired Student *t* test and Pearson correlation. Bland-Altman analysis was performed and the intraclass correlation coefficient was calculated to assess the agreement of both imaging methods. Values of p < 0.05 were considered significant.

## Results

All enrolled patients underwent CMR within a period of 1 month after 2DTTE, in 2 patients CMR followed 2DTTE within 2 months but follow-up 2DTTE confirmed stable LV dimensions in these patients. 3 patients had limited image quality on CMR and were excluded from further analysis. The baseline characteristics of the remaining study population are summarised in Table 1. Among the 38 patients included, 79% were males and the origin of MR was predominantly degenerative (92%). Severe MR, as assessed by the PISA method, was found in 45% of patients.

**Table 1:** Baseline characteristics of the study population

	**Study population (n = 38)**
***Demographic data***	
**Age (years)**	57 ± 14
**Gender (M/F)**	30/8
**BMI (kg/m**^**2**^**)**	25 ± 3
***Mitral valve analysis***	
**Degenerative MR**	35 (92 %)
**Rheumatic MR**	2 (5 %)
**Other ethiology MR (toxic)**	1 (3 %)
**Central / Eccentric jets**	19 (50 %) / 19 (50 %)
***Grade MR***	
**Moderate MR**	21 (55 %)
**Severe MR**	17 (45 %)

BMI = body mass index; MR = mitral regurgitation.

**Figure 3:**
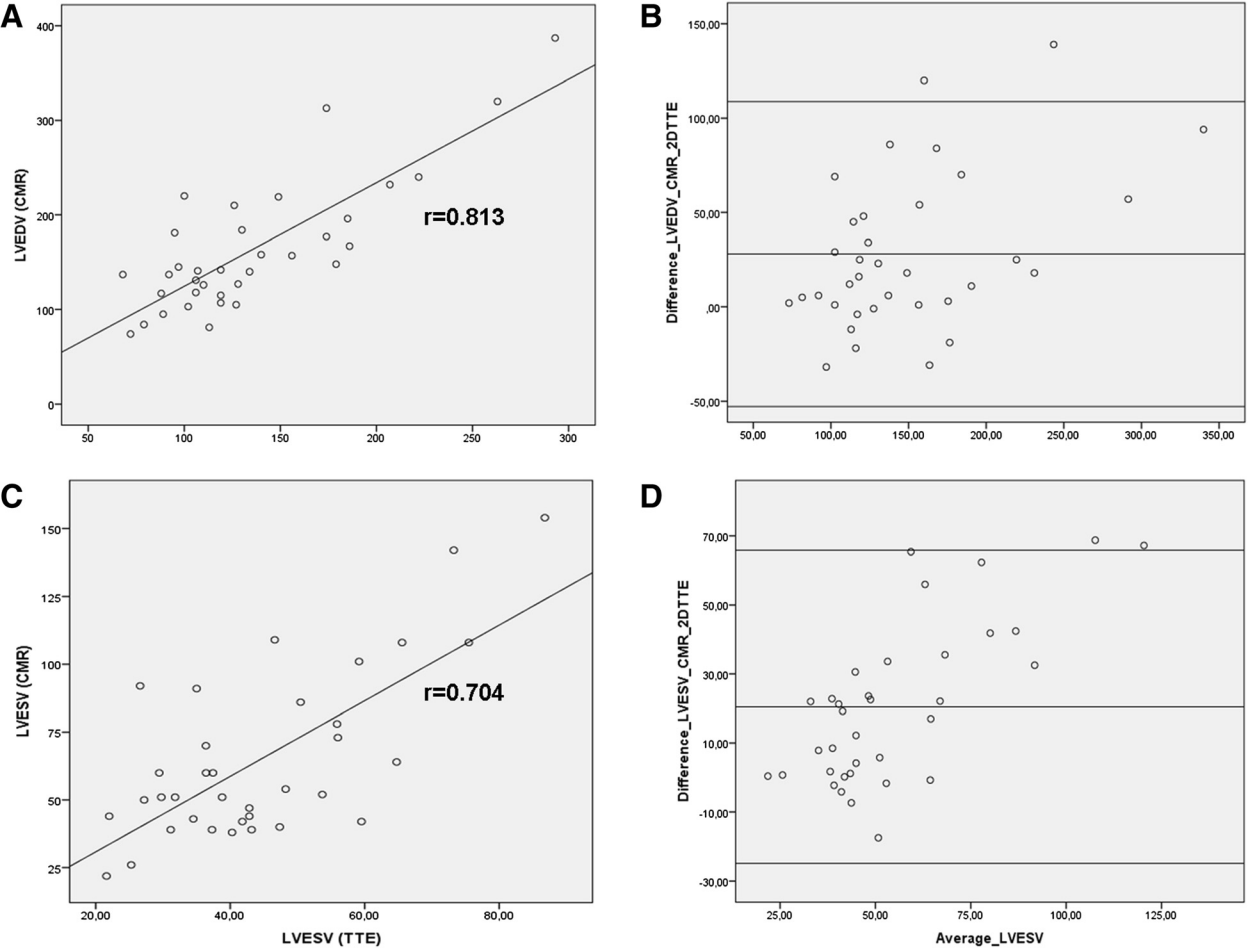
**Comparison of measurements of LV end-diastolic and end-systolic volumes by 2D TTE and CMR. A**. Measurement of LV end-diastolic volumes by both imaging methods showed a strong correlation by Pearson correlation analysis (r = 0.81, p < 0.00001). **B**. Bland-Altman analysis indicated general underestimation of the LV end-diastolic volume by 2D TTE in comparison with CMR (average bias +28 ml, 95% confidence interval -53 to +109 ml). **C**. Measurement of LV end-systolic volumes by both imaging methods showed as well a good correlation (Pearson r = 0.7, p < 0.00001). **D**. Bland-Altman analysis revealed general underestimation of the LV end-systolic volume by 2D TTE compared to CMR (average bias +20 ml, 95 % confidence interval -25 to +66 ml).

Comparison of measurement by 2D TTE and CMR regarding LV dimensions and parameters of MR severity are represented in Table 2. Measurements of linear LV dimensions by both imaging modalities were statistically similar (LV end-diastolic dimension: 53 ± 6 mm by transthoracic echocardiography vs. 53 ± 8 mm by CMR, p = 0.91; average bias -0.1 mm, 95% confidence interval -8.9 to +8.7 mm and LV end-systolic dimension: 36 ± 5 mm vs. 36 ± 6 mm, p = 0.95; average bias 0 mm, 95% confidence interval -5.7 to +5.7 mm). LV volumes were overall significantly underestimated by modified Simpson’s method in comparison with CMR, although LV end-diastolic and end-systolic volumes showed a strong correlation by Pearson correlation analysis (Figure 3A and C). Bland-Altman analysis confirmed general underestimation by 2D TTE of both LV end-diastolic volume (average bias +28 ml, 95% confidence interval -53 to +109 ml) and LV end-systolic volume (average bias +20 ml, 95% confidence interval -25 to + 66 ml) (Figure 3B and D). The LVEF was significantly overestimated by 2D TTE in comparison with CMR and there was no significant correlation between LVEF measurements of the two imaging modalities.

**Table 2:** Comparison of 2D TTE and CMR measurements of LV dimensions and MR severity

	**2D TTE**	**CMR**		**Pearson correlation**	
**LVEDD (mm)**	53 ± 6	53 ± 8	p = 0.9	r = 0.80	p < 0.00001
**LVESD (mm)**	36 ± 5	36 ± 6	p = 1	r = 0.85	p < 0.00001
**LVEF (%) teichholz**	64 ± 8	61 ± 7	p = 0.05	r = 0.26	p = 0.1
**LVEDV (ml)**	136 ± 52	164 ± 70	p = 0.0003	r = 0.81	p < 0.00001
**LVESV (ml)**	44 ± 16	65 ± 31	p < 0.00001	r = 0.7	p < 0.00001
**LVSV (ml)**	90 ± 39	99 ± 46	p = 0.06	r = 0.75	p < 0.00001
**LVEF (%) simpson**	67 ± 5	61 ± 7	p = 0.0004	r = 0.27	p = 0.1
**ERO/ARO (mm**^**2**^**)**	48 ± 25	42 ± 17	p = 0.1	r = 0.76	p < 0.0001
**RVol (ml) pisa**	69 ± 38	39 ± 27	p = 0.001	r = 0.38	p = 0.07
**RVol (ml) doppler volumetric method**	67 ± 33	28 ± 16	p = 0.003	r = -0.15	p = 0.6

MR = mitral regurgitation; LVEDD = left ventricular end-diastolic diameter; LVESD = left ventricular end-systolic volume; LVEF = left ventricular ejection fraction; LVEDV = left ventricular end-diastolic volume; LVESV = left ventricular end-systolic volume; LVSV = left ventricular stroke volume; ERO = effective regurgitant orifice; ARO = anatomical regurgitant orifice; RVol = regurgitant volume.

**Figure 4:**
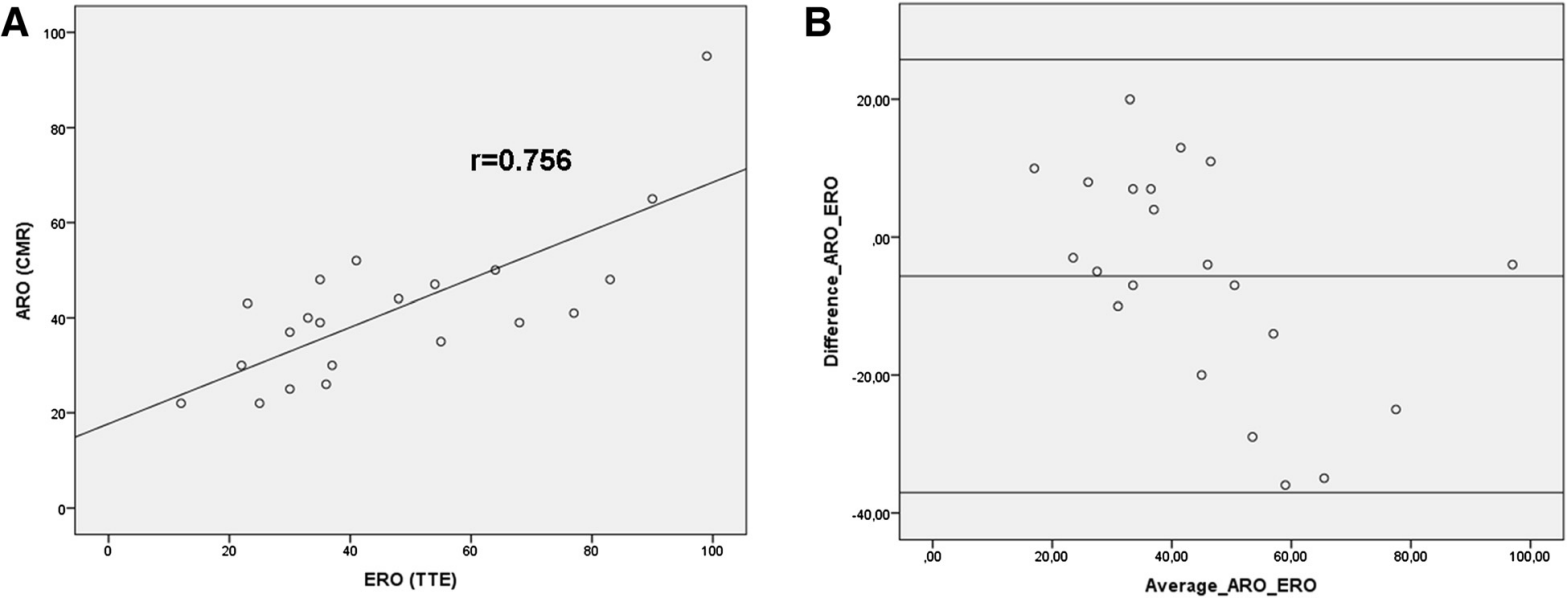
**Comparison of ERO measured by 2D TTE and ARO measured by CMR. A.** Pearson correlation analysis showed a strong correlation between 2D TTE PISA method and CMR planimetry of the ARO (r = 0.76, p < 0.0001). **B**. Bland-Altman analysis showed good agreement between both imaging techniques of the regurgitant orifice (average bias -5.7 mm^2^, 95% confidence interval -37 to +26 mm^2^), less accordance was observed in some patients with an ERO ≥50 mm².

Measurement of the ARO was feasible in 21 out of 22 patients (95%). ERO calculated by the PISA method with 2D TTE was similar to ARO measured by planimetry with CMR (47 ± 24 vs. 42 ± 16 mm^2^, p = 0.12). Pearson correlation analysis showed a strong correlation between both imaging techniques of the regurgitant orifice (r = 0.76, p < 0.0001) (Figure 4A). Furthermore Bland-Altman analysis showed good agreement between 2D TTE PISA method and CMR planimetry of the ARO (average bias -5.7 mm^2^, 95% confidence interval -37 to +26 mm^2^), less accordance was observed in some patients with an ERO ≥50 mm^2^ (Figure 4B). In addition, the intraclass correlation coefficient (=0.7, p = 0.0001) confirmed strong agreement between both methods to quantify the regurgitant orifice. By contrast, RVol calculated by either of the two 2D TTE methods was significantly higher in comparison with RVol obtained by phase contrast velocity mapping by CMR (PISA method vs. CMR: 69 ± 38 vs. 39 ± 27 ml, p = 0.001; r = 0.45, p = 0.07; Doppler volumetric method vs. CMR: 67 ± 33 vs. 28 ± 16 ml, p = 0.003; r = -0.14, p = 0.6). Assessment of regurgitant volume by PISA and Doppler volumetric method by 2D TTE was similar (74 ± 40 vs. 67 ± 33 ml, p = 0.6). Of note, 20% of patients with moderate MR according to ERO as assessed by the PISA method were reclassified as severe MR according to planimetry of ARO, likewise 20% of patients with severe MR by 2D TTE were reclassified as moderate MR by CMR.

## Discussion

### LV dimensions and volumes

An important finding of the present study is that LV volumes are significantly underestimated by 2D TTE in patients with moderate to severe primary MR in comparison with CMR, which is widely accepted as the most accurate non-invasive imaging tool to assess LV shape and sizes [10]. This finding might be of importance since adequate assessment of LV contractility and dimensions is crucial for clinical decision making in these patients. It is well known that LV volumes may be significantly underestimated (even up to 50%) by 2D TTE in comparison with invasive ventriculography or CMR in patients with normal and decreased LV function due to poor image quality, apical foreshortening and geometrical assumptions [5,11,12]. However, there are poor data about the degree of underestimation of LV volumes by 2D TTE in patients with primary MR with good echogenicity and without overt LV dysfunction; our data showed a mean difference of 28 ml regarding the LV end-diastolic volume and 20 ml regarding the LV-end systolic volume. Interestingly, there was a strong correlation between measurements of LV volumes by the two imaging methods, which may suggest that 2D TTE may still be useful to evaluate the progression of LV remodelling in a single patient. At present, guidelines use linear LV dimensions (LV end-systolic diameter) to define LV dilatation in primary MR as a criterion for surgery [4,13]. Our study showed very good agreement between 2D TTE and CMR regarding the measurement of linear LV dimensions. However, a recent CMR study by Schiros *et al.*[14] showed that the LV end-systolic diameter measured in the parasternal long-axis at the mitral valve leaflet tips may underestimate the LV volume due to spherical mid to apical remodeling. Further studies are needed to determine whether assessment of LV volumes has a benefit over LV linear dimensions for risk stratification in patients with asymptomatic primary MR.

### LV ejection fraction

Both Teichholz formula and modified Simpson’s method by 2D TTE seemed to overestimate LVEF in comparison to CMR. Furthermore, there was no significant correlation between both imaging modalities regarding quantification of the LVEF. However, our study population consisted of subjects with a preserved LV function and, as a consequence, a narrow range of LVEF (60-75%). Furthermore, 2D TTE is known to have a higher inter-observer variability than CMR, with a mean percentage of error that can be up to 10-15% [12] which implies that a large number of patients should have been included to detect any correlation between the two imaging modalities. Of note, 33% of our patients with severe MR and preserved LV function (LVEF ≥60%) by 2D TTE Simpson’s method had a mild decreased LVEF (50-59%) by CMR and therefore already have a class I recommendation for surgery [4,13]. These observations are in line with another small CMR study in patients with moderate to severe primary MR [15] and suggest that more accurate assessment of LVEF by CMR might be indicated in asymptomatic severe MR to determine optimal timing for surgery.

### Severity of MR

The PISA method with calculation of ERO and RVol is strongly recommended to assess MR severity [3]. However, several studies have used RVol obtained by phase contrast velocity mapping by CMR as a reference method to compare various 2D and 3D echocardiographic techniques to evaluate MR severity [6,8,16,17].

To our knowledge, only one study by Buchner *et al.* in 35 patients with mitral regurgitation described planimetry of the ARO and found a good agreement with calculation of the ERO by the PISA method [8]. In their study, 66% of patients had less than grade III mitral regurgitation, 66% had primary MR and 43% had a LVEF <50%. Their data showed that ARO was slightly higher than ERO, presumably because ERO represents the narrowest flow stream, which is located distal from the anatomic orifice. Our study confirms good agreement between the two methods in 21 patients with moderate to severe primary MR without overt LV dysfunction. We observed a rather higher ERO than ARO for most ERO values >50 mm^2^, although this finding has little clinical implications since most of these cases were graded as ‘severe’ by both methods. Thus, planimetry of the ARO by CMR seems to be a valuable alternative in patients with primary MR and poor echogenicity or eccentric jets which are difficult to assess with the PISA method.

In our study, RVol derived from CMR showed only moderate correlation with RVol obtained by the PISA method as well as with the Doppler volumetric method. Furthermore, RVol might have been overestimated by the PISA method in comparison to CMR, which is in line with a recent study by Hamada *et al.*[17]. This might be due to difficult determination of the - often dynamic - radius of the hemispheric contour of the flow convergence zone, especially in eccentric jets. By contrast, other studies found rather an underestimation of RVol by 2D and 3D echocardiographic techniques in comparison with CMR [18,19], indicating that in any case measurements of RVol by CMR or by 2D TTE are not interchangeable.

### Limitations

We did not perform more advanced echocardiographic techniques like 3D or contrast enhanced echocardiography, which are known to correlate better with CMR regarding LV volumes, LVEF and RVol [12,17-20] than 2D TTE. However, notwithstanding the well known benefits of these techniques, 2D TTE remains the most widely used imaging method in daily practice for the assessment of patients with primary MR. Furthermore, there was no comparison with invasive measurements of LV function or MR severity by angiography, but this latter approach is not any more recommended.

In most patients 2DTTE and CMR were not performed on the same date but within 1 month, which might influence the measurement of load-dependent parameters, in particular end-diastolic dimensions. However, we did not include patients with clinical or echocardiographic signs of heart failure and there were no patients with acute onset of symptoms during the study period. Furthermore, underestimation of LV volumes on 2DTTE was also seen in patients in whom both exams were performed on the same date.

## Conclusion

In moderate to severe primary MR without overt LV dysfunction, 2D TTE seems to significantly underestimate LV remodelling and overestimate LVEF in comparison to CMR. These findings might provide more evidence for routine assessment of LV size and function by CMR in asymptomatic primary MR in order to better define optimal timing for surgery. However, further studies are needed to determine whether the increase in LV volume is a more powerful predictor of the outcome than the end-systolic diameter. Moreover, the measurement of the regurgitant orifice with planimetry by CMR shows very good agreement with the validated PISA method by 2D TTE and thus may be a valuable alternative in patients with poor echogenicity or barely assessable eccentric jets.

## Abbreviations

MR: Mitral regurgitation; LVEF: Left ventricular ejection fraction; 2DTTE: Two-dimensional transthoracic echocardiography; CMR: Cardiovascular magnetic resonance; ERO: Effective regurgitant orifice; ARO: Anatomic regurgitant orifice; RVol: Regurgitant volume; PISA: Proximal isovelocity surface area.

## Competing interests

The authors declare that they have no competing interests.

## Authors’ contributions

CVDH is the first author, analysed off-line the echocardiographic data, and performed the statistical analysis. JM verified the echocardiographic data and statistical analysis and helped to draft the manuscript. LP and PL designed and coordinated the study in the University of Liege Hospital, acquired the echocardiographic data and helped to draft the manuscript. PB and LD acquired the CMR images in the University of Liege Hospital and performed the measurements of all CMR data. CDM acquired the echocardiographic data in the University of Antwerp Hospital. BP acquired the CMR images in the University of Antwerp Hospital. CV coordinated the study in the University of Antwerp Hospital and helped to draft the manuscript. All authors read and approved the final manuscript.
